# Application of Immersive Virtual Reality Interactive Technology in Art Design Teaching

**DOI:** 10.1155/2022/5987191

**Published:** 2022-08-27

**Authors:** Ying Ruan

**Affiliations:** Wuhan College, Wuhan 430212, Hubei, China

## Abstract

With the rapid development of economy and society, the integration of disciplines has become a key object faced by the whole society. The new characteristics of rapidly iterative technology and evolving theory of the digital age brought new challenges, especially for art and design teaching. At the same time, with the continuous progress of computer hardware level, all kinds of simulation technology constantly emerge, which also brought new opportunities for art design work. Art design teaching, as a systematic project, should use advanced teaching techniques with scientific theories. In the process of teaching design, how are all parts connected with each other, and what are the problems and needs of students and teachers? Through the analysis and research of these, we are aimed at finding ways and methods to solve these problems and needs, and to achieve the optimal teaching effect. The mode, technology, and methods of traditional art design teaching are getting more and more difficult to meet the needs of the society for comprehensive design art talents. Based on virtual reality technology and aimed at the art design teaching system, this paper studies the application of the immersive virtual reality technology in the design teaching practice. On the basis of the traditional design teaching mode, the integrated three-dimensional design teaching mode is put forward and verified. It can inspire students' creative inspiration in design teaching and guide them to immerse in learning and three-dimensional practice, constantly opening up creative thinking.

## 1. Introduction

In recent years, with the rapid development of science and technology, virtual reality industry has become gradually developed. Virtual reality is constantly integrating with cutting-edge technologies such as artificial intelligence, 5G, IoT (the Internet of Things), and Big Data. Science and technology promote the industrial application of virtual reality and give birth to new business and its ecology, such as Metasexes, Digital Twins, NFT, and other new technological concepts. With the popularization of 5G, many virtualization technologies are expanding to other industries, giving rise to richer mixed virtual reality applications. Education is one of the fields that urgently need new technologies like it to make virtual reality more intelligent.

Virtual reality technology is a popular network technology industry in recent years. It is also called “spiritual environment technology,” “virtual environment,” or “Cyberspace” [1]. Virtual reality technology involves many fields of technology, which is a combination of many technologies. It belongs to a big branch of simulation technology, including multimedia technology, graphics technology, computer perception technology, network technology, and so on [2]. Virtual reality technology [3] has four components [4]. The first is the virtual scene, which is a computer generated dynamic 3D image. Its effect is very realistic, making a feeling of immersive. The second part is perception, which includes all the perception that human beings should have, like vision, touch, sense of movement, and so on. These perceptual simulations enable virtual reality to create a sense of reality. The third part is the interactive function, including the Angle of view movement, hand-and-foot position movement, handle operation, and so on. The computer will get the user's behavior information, process, and respond to the information, and then, it will feed back to the user in the way of virtual reality. The fourth part is the virtual reality interactive equipment [5]. At present, it mainly interacts with the virtual scene through the helmet and handle [6]. The head theft provides three-dimensional display effect, and the handle is responsible for the interaction between hands and virtual objects. With the development of virtual reality technology, there will be more devices in the future to simulate various interactive functions. Therefore, virtual reality technology is in line with the concept of modern education development.

The industrialization upgrading of computer hardware is an important symbol of the rapid development of modern science and technology. It makes information technology, electronic information, and Internet technology widely applied to all aspects of life. The development of modern design is always two-way. On the one hand, it is increasingly internationalized and tries to make the design vocabulary transcend the limitation of nationality and culture. On the other hand, in order to maintain their own individuality, many designers try to find the source of creation from the cultural heritage of their own nation [7]. With the trend of global integration development, the national characteristics of art design are becoming more and more important [8]. The extraction of national traditional elements, and the reference and integration of foreign culture are the important task of today's designers, and also the important subject of today's higher art and design education. How to combine traditional culture and modern design style in the teaching of art design has always been the focus and difficulty of higher art design education [9]. The best way to solve the above problems is to constantly innovate the way of education. Science and art are ways and means for human beings to understand and explore the world. Their common foundation is human creativity. And the ideal science and art pursue is the universality of truth.

**Figure 1:**
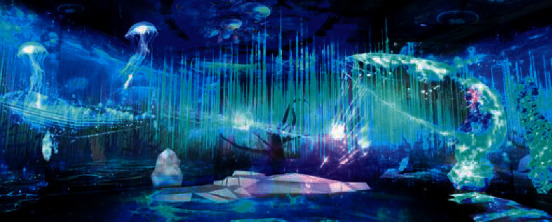
The stage effect of the immersive art drama The Wizard of Oz by China.org.cn.

With the update of technological media, the context of artistic creation is changing. This also prompts many artists to explore the artistic creation ideas with multimedia technology as the media, and carry out integrated research on technology, modeling, concepts, and other topics. The mainstream language of contemporary art has been formed by the display of various media; the integration of high-tech, behavior, and skills; and the collection of various kinds of art [10]. As a medium, virtual technology intervenes in artistic creation and combines the performance and characteristics of art.  Figure 1 shows the new art form—virtual plastic art. Virtual plastic art, as an important element of contemporary art, transcends the limitation of traditional artistic expression [11]. Through the combination of art and technology, it becomes a new way for people to know and experience the world [12]. It can not only simulate the shape and beauty of the real world but also enrich people's imagination and creativity. It is leading the direction of art development as a form of artistic creation by means of multimedia and virtual technology. And it is bound to become the mainstream culture in the future.

The rapid development of virtual reality technology and the continuous emergence of various simulation technologies have brought new opportunities for practical design work. And this fact also caused us to think about the combination of environmental art design teaching and virtual reality technology. In the increasingly intense market competition environment, how to improve the presentation and experience quality of art design works has become a common concern of experts and scholars. And for schools offering art and design majors, how to improve the practical teaching quality of art design major is also the direction of teachers' continuous exploration. Research results show that art design in the new era needs more experience and changes to meet the emerging demands brought by economic development [13]. With the popularity of arts in public life, researchers began to sort out and analyze the experience and design strategies of arts education. The study of art education mode and management strategy, to combine new science and technology with traditional education, is the most correct decision.

## 2. Related Studies

### 2.1. Development of Virtual Reality Technology

Limited by the development of communication, computer, and simulation technologies, VR technology has experienced a long process from the concept to the basic perfection of the theory. The development of virtual reality began in the 1920s [14]. And the development of computer field led to the improvement of simulation, virtual reality, and other related fields. Through the efforts of many practitioners and scientists, virtual reality technology has developed rapidly and now has been extended to various fields. The emergence and development of VR technology has greatly benefited from the help of scientific theories, especially the theoretical development of computer technology [15]. The development of computer technology has directly promoted the progress of VR technology [16].

In 1965, Sutherland, an authority in the field of computer graphics, wrote a paper known as “The Ultimate Display” [17]. In this book, he firstly proposed a simulation idea. Then, the prototype of virtual reality was born. He plans to use virtual simulation to enable the experiencers to have a perceptual experience similar to the real world. He also envisions experiencers interacting with virtual objects created by computers, by using computers to process incoming information and process feedback. The virtual reality technology in that time was in its infancy, and the technical thought was immature. Moreover, the key discussion of some scientists opened the door of virtual reality and led the future development.

At the end of last century, the field of virtual reality ushered in a breakthrough, and several complete virtual reality systems came out. Although various theoretical articles on VR have been published, many countries have also carried out relevant practical research out of the support of these theories. However, due to the low popularity of the Internet at that time, many technical difficulties have not been broken through. The application research of VR is more inclined to the enterprise side, and there are few products that actually enter the consumer field. In 1983, American academic research institutions formulated and implemented the Simulation Network plan, which greatly promoted the development of virtual reality [18]. The results of the program have had a profound impact on virtual reality. In 1984, VR display technology achieved a landmark achievement. NASA simulated and analyzed the astronomical data of the Solar System and successfully simulated the virtual model of the Solar System [19]. A large number of successful cases continue to promote the development of virtual reality technology. However, in the 20 years since 1990, as theoretical research has gradually slowed down, the VR industry has not burst of vitality as originally expected.

After 2000, with the rapid development of network technology and the birth of computers with powerful 3D computing capabilities, high rendering quality, and transmission speed, virtual reality technology has also changed from an idea to a reality [20]. Virtual reality technology has very broad application prospects in the fields of economy, military, education, entertainment, and so on. In February 2008, the National Academy of Engineering released a report [21]. It stated that virtual reality technology has also become one of the 14 major challenges facing engineering in the 21st century, like other technologies—easier access to solar energy and fusion energy. In order to strive for the commanding heights of virtual reality technology, governments and large companies in developed countries have invested huge amounts of money in research and development in this field. The fundamental purpose of VR technology is to realize human-computer interaction in 3D mode. Therefore, academia and many manufacturers generally take immersion, interaction, and imagination as the basic criteria for designing VR systems [22]. In recent years, with the vigorous development of artificial intelligence-related theories, intelligent has also become another significant feature [23].

In recent years, countries around the world have listed relevant policies to promote virtual reality technology. And various national scientific research funds have taken the research of virtual reality technology as an important direction. At the same time, some key laboratories have also begun to invest time and energy in this field, and already got some results. The teaching means provided by VR technology that can make the classroom environment more diverse. The national administrative authorities are in charge of education policy. The Ministry of Education, the Ministry of Industry and Information Technology, and the National Development and Reform Commission all have active policy guidance on the development of virtual reality. As far as the application of virtual reality is concerned, the more attention is paid by the competent authorities, and the more in-depth virtual reality technology will be applied in teaching. The Chinese government has launched a program to support intelligent hardware enterprises to meet education needs. And it also apply intelligent hardware technologies in distance education, smart classrooms, virtual classrooms, and online learning, so as to improve the intelligence level of education. Combined with the development of intelligent hardware products, high-quality teaching resource libraries should be built. It can connect online and offline education resources, expand the coverage of high-quality education resources, and promote education equity. The special action proposed for virtual classroom development is conducive to the construction of high-quality teaching resources.

### 2.2. Teaching Research Based on VR

Education informatization promotes education equality and justice, and continuously reduces regional differentiation. It plays a very important role in improving the quality of education all over the world. The application of virtual reality and augmented reality in education conforms to the trend of global information development and creates an atmosphere of universal learning, autonomous learning, and lifelong learning. The involvement of virtual reality and augmented reality in the field of education of the Internet era provides new forms of interaction for learners.

Art design is an ancient humanities subject, and it is challenging to apply technology in art design teaching [24]. Through a large number of studies, scholars have found that the research in the field of visual space is most suitable for the use of immersive virtual reality technology [25]. The representation of visual space is to construct a visualized scene in cognition. In this scene, the visual subject enters with it and transforms that moment into the present moment. Like creating a virtual scene, the character places himself in the scene, and virtual reality technology achieves this perfectly. With computer technology to create a space environment that simulates reality, students can engage in certain environment by using a variety of sensing devices. Students can also carry out perceptual operations on various objects in the virtual space, so as to obtain real experience and experience. This is a computer technology that enables students to communicate directly with the virtual environment [26].

Art and design education is a kind of creative education, which is different from other subjects [27]. The difference between arts and other disciplines lies in its openness and diversity. There is no constant standard for aesthetic art [28]. And the ultimate orientation is the pursuit of possibility and creativity. But in the teaching of art design, its main goal is to exercise students' mental thinking, work creation, hands-on practice, artistic aesthetics, and other basic abilities [29]. The development of modern society requires the cultivation of artistic literacy as the core, supplemented by practical activities and humanistic structures. Moreover, teachers should pay attention to cultivating students' independent and distinctive personality and characteristics in the specific teaching process. On the one hand, this will train students' imagination and train them to develop art creation, so that they form their own personality quality in the process of creation. On the other hand, it promotes students' free and creative thinking. Scholars' research shows that creativity often occurs in a relaxed and focused environment. That is, we provide students with a virtual environment and scene reproduction, add human-computer interaction according to the teaching content, and use the arrangement of exploratory tasks to carry out learning. Immersive environments can often inspire and stimulate learners' creativity, and then, relatively independent space will enhance their concentration.

Although China began to study virtual reality technology not early, but with the rapid development of computer field in recent years, it also drives the development of virtual reality technology. In recent years, virtual reality has come to the fore with a wide range of commercial projects. With the continuous maturity of technology, virtual reality space art becomes a combination of sculpture, painting, video, and other media of the new art language form. Virtual reality has a wide range of applications, but it is still in the exploratory stage. Its technology involves electronic technology and requires the application of visual perception, physiology, psychology, ergonomics, and other disciplines. It needs to be promoted from technology level to art level. So its research and practical application in the field of art is what it needs to explore urgently.

**Figure 2:**
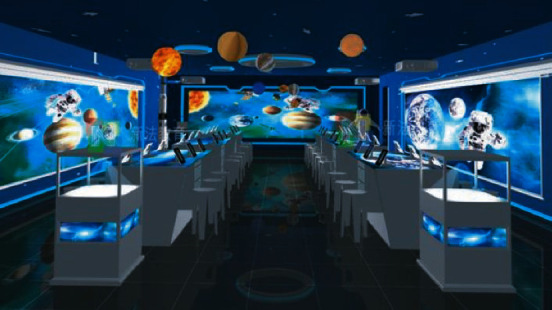
Immersive VR classroom of Shenzhen new law education.

With the gradual improvement of the basic theory of science and technology, the development of applied science and technology has entered the stage of exponential rapid iteration. Virtual reality technology has been gradually applied in the teaching of many subjects. And it gradually shows signs of changing the traditional education mode. At present, many educational and scientific research institutions around the world have carried out teaching practice and exploration research using virtual reality technology, as shown in Figure 2. It can be seen from these studies that virtual reality technology has great advantages in promoting students' learning. As a new auxiliary means of design teaching, virtual reality technology enables the performance of teaching content more vivid than before and also can effectively create a teaching environment to follow the development of technology.

The appearance of virtual reality technology will have a profound influence on the traditional design curriculum. It will change the past and present concept and mode, and break the time and space limit of design teaching. And it will make the development of design teaching present infinite possibilities. At the same time, we should also realize that the popularization and application of virtual reality technology in teaching is still facing many challenges. As a pioneering educational technology, it is necessary to explore the potential advantages and challenges of virtual reality application, and then provide theoretical support for further experimental research.

However, virtual reality technology still has technical limitations, such as display technology with lower resolution, images with obvious delay, and unnatural human-computer interaction methods [30]. Moreover, all these cannot limit it as [31] the best visual space cognitive research tool. And with the development of technology, these defects will be gradually solved [32]. The application of this [33] technology opens up a broader space for the development of design teaching. With the continuous development of science and technology to other fields, virtual reality technology has become a relatively mature teaching means [34]. Numerous research results can provide design guidance for the development and application of virtual reality teaching technology. Among them, graphic theory is the formal composition principle that should be followed in the construction of virtual reality teaching system. All these can scientifically promote the application of virtual reality technology in design lessons.

## 3. Art Design Teaching Mode Based on VR Technology

**Figure 3:**
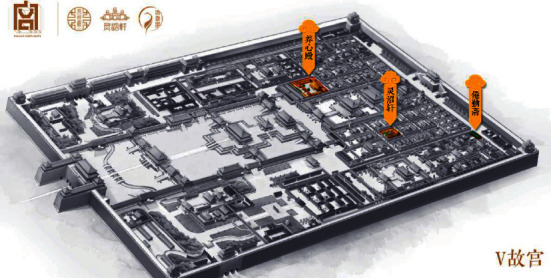
Main interface of virtual exhibition hall of Beijing palace museum.

Art design forms are changeable, mainly in the field of visual perception [35]. The art application based on VR technology first started in museums, such as the virtual exhibition hall of the Palace Museum as shown in Figure 3. The teaching types based on immersive virtual reality technology are more complex and diverse. For example, the relativity, integrity, and organization of visual perception, classroom experience, and knowledge acceptance have a very close relationship. How to sum up a number of visual perception organization principles such as simplicity and continuity? How to apply these visual organization principles in art design? All need to be considered. Through the design of teaching practice based on immersive virtual reality technology, we can see that there are many differences between design teaching and design creation practice. Design creative practice activity is an independent creative designer. Guided by their formed worldview, designers use appropriate creative methods to gain experience through observation of real life, and then conduct research, analysis, selection, processing, and refinement of various materials. And finally we present the design works. We come out and make each learner feel more deeply about these works of art.

### 3.1. Basic Teaching Model

In this process, the designer's thoughts and actions can be relatively independent, pure, and internalized creative activities. Design teaching is an externalized group behavior and a systematic project. Teaching design should use scientific theoretical guidance and advanced teaching techniques. By analyzing and researching how the various parts of the design teaching process relate to each other, its problems, and needs, and then find ways and means to solve these problems and needs. In the end, the best teaching effect can be achieved. In the teaching planning of design course, the mode of design teaching should be established first. Design is called art because it should not only adapt to the development of the times but also lead the trend. Therefore, in order to make modern art design and innovation closely linked, we must make full use of virtual reality and other scientific and technological design teaching mode. This paper will be based on the basic framework of subdesign teaching activities. In the design teaching work, every professional teacher will choose some suitable teaching modes for design teaching. The mode of design teaching is not immutable, but is constantly breaking through and changing with the development of basic theory and applied technology.

The traditional teaching mode achieves the final teaching goal through three stages: modeling foundation, design foundation, and professional design. In terms of curriculum setting, each stage is divided into several independent courses of its own system. The courses on the basics of modeling include sketching, color, and sketching. And the courses on the basics of design include the basics of decoration, plane composition, color composition, three-dimensional composition, and computer-aided design. In the professional design stage, the course settings are distinguished by types, such as advertising design, display design, environmental design, and industrial design. In this mode, it can be seen that the curriculum setting is based on design types or design elements, which is convenient in teaching implementation. However, with the development of the times, in the process of cultivating comprehensive innovative talents, this teaching mode gradually shows some shortcomings and disadvantages. Each stage of “three-stage” design teaching mode is relatively independent and systematic. In the process of learning, it is difficult for students to integrate these fragmented courses. Students cannot grasp the courses of each stage as a whole. This means that it hinders the full play of creativity and prevents them from creating comprehensive design works and practicing design activities.

At present, the integrated design teaching mode occupies the main teaching subject. It not only conforms to the law of design teaching but also accords with students' research and learning of visual perception-related theories. Other scientific and technological means are also expanded based on this. In design teaching, we should first grasp the integrity of each course with different forms, types, and styles. In the integrated design teaching mode, the setting of the course requires a large number of relevant teaching resources to be reorganized, focusing on key structural systems such as form, type, and style. Form serves as the main carrier and medium to convey visual language. Type integrates professional design and forms a series of design practice subjects in combination with social and industrial division of labor. Style is to integrate those stable characteristics in the history of design, to summarize and study, and to grasp and apply it on the whole.

### 3.2. Art Design Teaching Mode Based on VR

Virtual reality technology is an important auxiliary means of integrated design teaching because of the rearrangement of relevant design teaching resources needs to involve many fields, such as philosophy, history, mathematics, physics, geography, and so on. It is necessary to make many changes in the learning and use of resources. And students are easy to jump out of the teaching scene whenever this time. In the virtual reality classroom, resources in various fields are fully integrated. And students are completely immersed in the virtual situation, excluding the interference of things in the real environment so that they can broaden their horizons and have a more solid knowledge base. The immersive advantages of virtual reality can be fully played here. For example, in the design teaching, it is necessary to refer to the design or style of a real building. Teachers can collect and integrate relevant information in the virtual reality teaching system and lead students to enter the Old Summer Palace in virtual reality. Students can also dress up in ancient costumes, enter the building, and even travel through the past and present by adjusting the timeline to observe the design, construction, use, and demise of the entire building.

Virtual reality technology can provide necessary technical support for the smooth implementation of design teaching in the course of designing the key structural system curriculum. For example, in the design teaching series of form system, students need to learn and analyze single page, folding, three-dimensional, space, and other forms to achieve design practice. Through the interactive nature of virtual reality technology, students can easily perceive the difference and connection between various forms. And they can skillfully apply the transformation of various forms in the design practice and gain more the design ideas.

In the design teaching of type system, the concept of virtual reality can enable students to better observe the social industry division of labor and the application scenarios of design works such as signs, packaging, and displays. We make them to experience the real natural feeling. We enable practical design practices better meet the needs of users. In the design teaching series of style system, the multisensitivity of virtual reality technology can combine multisensory information such as vision, hearing, touch, and smell. With the help of virtual reality technology, teachers led students into the interior of real buildings and listened to ancient music combined with real scenes. Students can quickly develop a deeper understanding of the learning style and have their own unique experience.

Teaching in the virtual teaching environment constructed by virtual reality teaching technology can fully mobilize all the sensory systems of students through interactive methods such as watching, listening, and touching. It gives them a sense of presence that is significantly higher than other teaching methods. This sense of presence is a special psychological experience obtained by the interlocutor through virtual reality technology. In the virtual reality teaching system, students are completely immersed in the learning environment created by virtual reality teaching technology. The virtual reality teaching system is highly interactive. Students are immersed as participants, as creators. They are no longer looking at a computer screen and passively receiving, but become a part of it. Psychological immersion is the illusion of being present in an unreal environment, thereby deepening the all-round three-dimensional experience.

With the support of virtual reality teaching technology, students can no longer passively accept what they have learned, but integrate into it through various ways of participation. This process includes the generation, development, and change of the whole event to make the learning process and life closer. In this learning scene, students are not only able to observe works of art from multiple perspectives but also to influence their own visual experience by interacting with the virtual reality system. Compared with one-way reception of learning content, such a two-way interactive experience can stimulate students' interest and deepen their understanding of learning content. It can also take the initiative to apply knowledge points in many places to realize the transfer of art learning theory. Through flexible bidirectional interaction, virtual reality technology breaks the one-way transmission channel of knowledge in traditional teaching and realizes direct interaction between students and learning content. Teachers and students can also play roles together in the virtual environment to promote the teaching process and form a harmonious, interactive, and cooperative teaching relationship.

## 4. Application of Immersive VR in Art Design Teaching

### 4.1. Immersive Virtual Reality Art Design Teaching System

New media art has various forms of expression. Art design and Internet technology are the inevitable result of the development of the times. People's dependence on science and technology in production and life makes people unable to part with the screen anytime and anywhere. Thus, it inevitably results in the inseparable relationship between new media art and network interpersonal relationship. Art design teaching must grasp the psychology of students, considering that people spend most of their time in front of the screen to communicate. The situation and works of new media art are important contents to support interpersonal communication, such as images, videos, and other video materials. All these are popular communication contents in this era, especially for students surrounded by computer technology.

**Figure 4:**
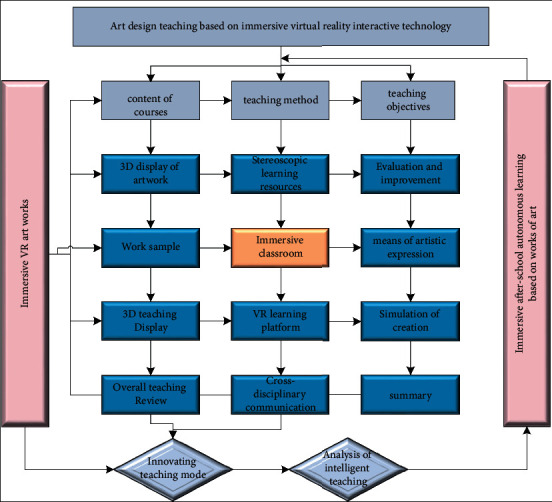
Teaching system diagram of immersive virtual reality art design.

Students passionate about network interpersonal interaction and play with various new media digital products. At the same time, they are using new media art extensively in interpersonal communication. They send videos and pictures through the short video platform. Some creators through virtual reality technology even play games, watch animations, etc. All are the teaching and dissemination of new media art. The teaching system flow of art design major based on immersive virtual reality interactive technology is shown in Figure 4. We comprehensively consider the characteristics of virtual reality technology and the learning scope of art design. We divide the research into three aspects: teaching content, methods, and objectives. Each aspect is progressive, continuing to extend downward according to the virtual reality-based learning progress.

The teacher first converts the art design content into three-dimensional VR works through virtual reality technology. Then, students can intuitively appreciate the works through computer VR equipment. Students interact with works and artists physically and psychologically. They experience artists' creative ideas through the process of art creation and trigger viewers' own thoughts and emotions. In the process of appreciation, students continue to learn more details of the works and learn creative methods through communication and interaction with teachers. The essence of this teaching method of art presentation using virtual reality technology is to let students experience its unique external characteristics. Through professional equipment, the three-dimensional art presentation is restored and recorded, and finally produced into a sophisticated panoramic video. Students can experience the reality and shock brought by art in the virtual world through immersive virtual reality experience through VR somatosensory equipment. Students increase learning interest and improve learning efficiency.

With the continuous popularization of 5G technology, live VR art teaching will become more abundant. Through three-dimensional teaching of VR art works, teachers can not only fully understand students' learning status but also stimulate students' enthusiasm for art creation. In this lively teaching process, students will no longer be affected by their own learning ability, because VR art is three-dimensional for everyone.

Technology applications are everywhere in the Internet era. Art teaching based on this kind of immersive VR can also be well matched with intelligent digital analysis. It is helpful for teachers to improve teaching methods by analyzing students' learning videos and browsing records. In a word, virtual reality technology is used to present art teaching, which makes art appear in a new form in front of students. It completely breaks the situation of art being refrigerated. It enables students, academia, and the government to pay attention to the protection, inheritance, and development of original ecological art. It also enables many traditional arts with complex performance forms and on the verge of extinction to create brilliance again, to enter the field of vision of the public, thus attracting widespread attention.

### 4.2. Immersive VR Art Design Teaching Process

Art is the product of civilization summarized by the long-term development of human society, and it is bound to have a relationship with the characteristics of the times in any period. In this era of rapid development of science and technology, modern science and technology and traditional art and culture are bound to collide and produce sparks. The communication and presentation methods of the art world will be overturned. And the expression form of “immersive” art was born under such an era background. The integration of network resources into the teaching of new media art aims to cultivate students' ability to acquire learning resources, apply analysis, and self-learning ability. It enables students to have the information literacy of new media art. Then, they can be good at using information technology, active learning, exploring, and researching. And it can promote lifelong learning.

Art design includes digital photography, digital video, computer art, design art, and other works of art. Such works of art are derived from virtual network technology. Unlike traditional painting, sculpture, and craft works, which are derived from real objects, they can be appreciated through physical display, touch, and experience. New media art works must be displayed through the network technology platform. So, in the new media art teaching appreciation link, it is necessary to use the network platform to display. Teachers can use VR display works for teaching appreciation and take the massive art resources on the website as the main source of students' appreciation. When teaching computer painting appreciation, teachers should use the content of computer painting exhibition website or competition website for students to appreciate their works.

VR art creation needs to be completed through computer digital technology. Computer software is a tool and material that must be familiar with when creating art works. Students need to master computer software to carry out practical operation, just as Chinese painting learners must be familiar with painting tools before learning landscape painting. During the demonstration teaching of computer software operation, teachers can create network platforms or make use of existing live broadcasting platforms to carry out online teaching. Online live teaching, such as short videos, is highly interactive. And teachers can adjust their speed and content difficulty in time according to the feedback of students. Students can also watch teachers' teaching while carrying out practical operation drills. If they have any questions, they can timely communicate with teachers through barrage. Teachers can answer questions online without any time difference. This kind of class all people can participate in the discussion with a high level of interaction.

Traditional art works can be displayed and communicated through art galleries, art exhibition areas, art squares, and other venues. But new media art works need more scientific and technological products to be displayed. For a small number of new media art exhibitions, such as schools, classes, or individuals, the most ideal venue should be the live broadcast platform. Teachers should move the display of students' works to the Internet. The website can achieve cross-region, cross-time, and space on the display of students' works, and encourage students to conduct interpersonal interaction learning on the website.

Network interpersonal interaction is very important in the evaluation of new media art teaching, and it is also the focus of immersion learning research. The self-learning mechanism in network interpersonal interaction helps to improve people's self-satisfaction and promotes active initiative in doing things. VR technology will be used to introduce network interpersonal teaching, and its website will be used as an interactive platform for teaching. Teachers and students will learn and communicate through the website resources. Students upload the completed new media art works to the online platform for display, using the evaluation in human interaction as a reference. At the same time, teachers can comprehensively consider the offline learning situation and finally make a reasonable teaching evaluation.

### 4.3. Immersive VR Art Design Teaching Method

On the one hand, network data platform has massive resources and information, and its sources are extensive, throughout the world. On the other hand, the Internet is rich in content, covering all fields of art. Through the Internet to provide learners and teachers with rich sources of knowledge, teachers use VR immersive teaching mechanism, create, or provide an interactive learning platform or appropriate education website. Teachers provide online multimedia teaching resources or online webcast teaching. The teacher creates a VR link on the Web. On this link, students' works of art can be displayed. Teachers and students can evaluate and communicate with each other anytime and anywhere after class. Extending the breadth of classroom learning time also allows learners to record their learning. At the same time, online teaching can be carried out through network broadcast or online teaching platform to enhance classroom interaction and timeliness of evaluation. Network interactive teaching has broken the traditional time and space restrictions. With the advancement of education informatization and the continuous development of network interactive platform teaching technology, it will become the mainstream teaching method in the new era.

Through the establishment of the school's own VR website, students are encouraged to upload their own art works. First of all, new media art itself is inseparable from the Internet, and the online exhibition platform is just like the art museum and art exhibition area in real life. The exhibition hall of new media art is the network platform, so students need to display and exchange their works, and constantly learn art details through VR. Displaying students' works can also effectively promote students' motivation to learn. In order to gain recognition from others, students must constantly improve their works of art. A website enables commercial transactions while presenting one's own work. If you put your favorite works on the Internet, people in need can buy or download them, thus gaining economic benefits. This is undoubtedly the motivation to promote students' learning.

In addition to teaching based on VR websites, virtual reality teaching based on 5G desktop is also a very important aspect. In the desktop virtual reality system, the computer screen is the window for students to observe the virtual world. With the help of professional teaching software, students can learn knowledge and solve problems in the design process. Students watch the three-dimensional effect of virtual 3D objects or scenes through the display. This will make students interested in learning. With the rapid development of 5G technology, desktop virtual reality systems are becoming more and more immersive. In the teaching work designed at this stage, a lot of time is almost completed on the computer. Students participate in the production model to make indoor and outdoor environment roaming map. And this can realize the computer simulation of real needs and can give students the most intuitive feelings.

## 5. Analysis of Immersive VR Art Teaching

### 5.1. Immersive VR-Based Art Design Professional

Art design teaching is not only an art but also a wide range of disciplines. It mainly includes product design, environmental design, graphic design, multimedia design, and so on. Its aesthetic standard is different from that of traditional art, which is always changed by many factors. Artistic design aims to serve the designer's own humanistic quality and aesthetic experience. Artistic design is actually the embodiment of the designer's own comprehensive quality, such as creative ability, perceptual ability, and imagination ability. As a teacher of art design, we have to use a variety of teaching methods to practice boldly.

Product design is a thinking method of solution planning to solve problems. It specifically solves the development and improvement of physical objects or tools. Through the specific planning process to make the product to get a good solution, the form of good product design reflects the economic, technological, and cultural characteristics of an era. Therefore, product design is of great significance. If a product lacks good design, production will cost a lot of money to adjust and change the production equipment and processing materials. On the other hand, a good product design will be easy to manufacture and low production cost, so as to improve the comprehensive competitiveness of the product. The application of virtual reality technology to product design teaching will stimulate students' creativity, and it can also be produced by showing product details so that students can clearly understand the product production process as a whole.

The curriculum of environmental art design covers not only interior design but also landscape design and architectural appearance design. Interior design can be subdivided into display design, bedroom design, office space design, catering design, hotel design, and so on. Environmental art design focuses on practicality and covers a wide range of subjects, including architecture, urban planning, environmental science, psychology, design, aesthetics, and so on. Virtual reality technology can be used in environment design teaching. Indoor scenes can provide roaming for them. And outdoor scenes can simulate natural scenes and imaginary scenes to stimulate students' design and creation ability.

Using virtual reality technology, the teaching of environmental art design is more intuitive. Virtual reality technology can simulate various types of spatial environment data, so as to apply it in teaching. And virtual reality technology is still constantly improving equipment and strengthening processing capabilities. It is hoped that it can finally be better applied to the teaching of environmental art design and make the teaching system of environmental art design more perfect. Thus, it can fully tap the potential of students and reflect the subtlety and complexity of environmental art design. The teaching of environmental art design can obtain rich teaching materials through virtual reality technology, so as to continuously develop its research theory.

Graphic art design itself is a relatively high requirement of professional disciplines. So only through effective practical teaching, it can be applied to the knowledge of graphic art design. Graphic design is based on vision as a means of communication and expression. It can be presented in a variety of ways, such as combining symbols, pictures, words, and other elements for visual representation. Graphic designers use ideas to create objects they want to express. Its major generally uses computer software professional skills to complete creative planning for design objects.

VR teaching improves the practical ability of graphic art design for students, enriches classroom teaching methods for teachers, and shortens the distance between schools and society. Therefore, the practical teaching of graphic art design has a certain value of the times. The practical teaching of graphic art design is the inevitable trend of the new situation and the development of new classroom. Meanwhile, the teaching of graphic art design is also the result of the interaction of various disciplines. Therefore, the traditional teaching of graphic art design can no longer meet the needs of the society for design talents. At the same time, the practice-based teaching method of graphic art design has been explored. By constantly improving students' practical ability of design, students can be more adapted to the dynamic development of graphic art design in the society. In short, interactive teaching based on VR is the inevitable trend of graphic art design development.

### 5.2. Three-Dimensional Teaching Examples

Art design requires all-round observation. So, we gradually experience the spatial thinking of VR teaching through the 3D original car suggestion model and the whole process, to achieve the ability to independently analyze and make models. The experimental course construction of art design emphasizes the exploration of teaching. It puts forward new requirements for the teaching content and course summary of teaching methods.

**Figure 5:**
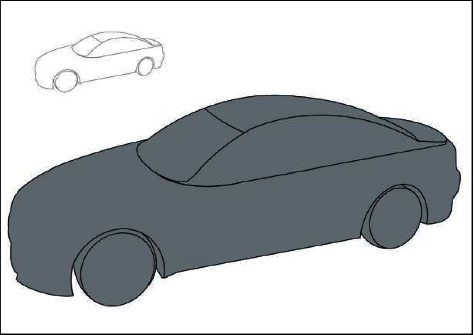
Original car 3D stage 1 basic line drawing.

Art design teaching based on immersive virtual reality starts from the perspective of students themselves. In this mode, teachers can find problems existing in students from different perspectives and then to help solve problems from the perspective of curriculum design with innovative perspectives. In the course of art design teaching, this paper takes product design model making course as the interface between VR technology and design teaching. Taking Figure 5 as an example, basic art design starts with points and lines, and sketches the primary model through lines. The lines of the basic works of art should be simplified as far as possible with few structural points. And the symmetrical ends should be kept even as far as possible, so that the subsequent asymmetric ends may have a stronger three-dimensional sense.

**Figure 6:**
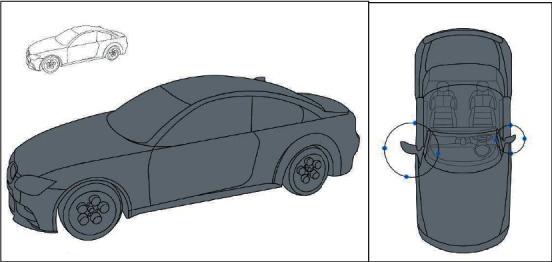
Original car 3D phase 2 intermediate line drawing.

As shown in the left picture of Figure 6, although the outline of lines is still displayed, the existence of surfaces can be obviously felt through the superposition of lines. On the basis of ensuring high-quality splines, the surface is generated. And the complete modeling of the surface is constrained by distance. The greatest significance of virtual reality teaching lies in multi-angle observation, as shown in the picture on the right of Figure 6. Through multi-angle observation, teachers find problems existing in students' creation process. As is shown in the figure, there is no problem with the basic construction of art design from the perspective of students. However, the teacher found basic design errors in other angles (the left and right ends of the work did not reach the design symmetry). The most important thing is to let students feel the three-dimensional idea of this design through VR, rather than simple visual errors. This intuitive feeling cannot be achieved by traditional education.

**Figure 7:**
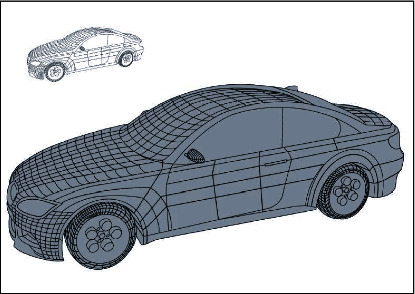
Oil painting style rendering simulation.

As shown in Figure 7, under the guidance of teachers, the final result not only shows the current visual effect but also unique perspectives from other perspectives can still appear in students' memory. The current teaching of new media art needs the support of interactive thinking and interactive platform. Interactive thinking refers to the interaction between people, people and machines, people and the network, and people and the environment. In the new media teaching, everyone should have highly frequent interactive thinking to participate in learning so that the teaching will be more effective. As is shown in the picture, man and machine need a place for communication and interaction, that is, an interactive teaching platform. Teachers and students can have a dialogue on this platform, and teaching can be realized. The charm of VR interactive teaching course lies in displaying three-dimensional visual colors. And the design has carried out a detailed and comprehensive expression in this aspect.

## 6. Conclusion

Art design teaching is based on immersive virtual reality technology and computer technology as the carrier. Through the combination of virtual and physical interface operation and interaction, students can experience the series of design processes to the greatest extent such as space secondary design and interface form design. Students can fully experience the different atmospheres of different designs in the immersive creation process. Experience the good and bad changes made by different design changes, so as to master the theoretical knowledge of design unconsciously. Art design is a practical science course. Apart from classroom teaching, students are required to go to the field for special practice. However, these nonlinear learning activities have poor teaching effect. VR interactive learning can realize real learning and creation in the classroom. Students can get knowledge feedback from teachers in time, and the teaching effect will be better. This kind of teaching method is also seeking a breakthrough in the traditional teaching mode.

With the help of virtual reality technology, the heterogeneity and isomorphism theory of art design major is applied in design teaching. This can better enable students to grasp the beauty of design art from an overall perspective, and integrate relevant elements into design practice. In this new model, teachers can help students better understand the isomorphism of form and meaning. And we stimulate students' artistic creative thinking. This can transfer the knowledge gained from the immersive experience into the design planning. It can make the development of design teaching to show infinite possibilities.

## Data Availability

The experimental data used to support the findings of this study are available from the corresponding author upon request.

## References

[1] Yang Y., Wang H. (2016). Application of virtual reality technology in automobile teaching. *Science and Education Guide: Electronic Edition*.

[2] Olshannikova E., Ometov A., Koucheryavy Y., Olsson T. (2015). Visualizing Big Data with augmented and virtual reality: challenges and research agenda. *Journal of Big Data*.

[3] Biocca F., Delaney B. (1995). Immersive virtual reality technology. *Communication in the age of virtual reality*.

[4] Alqahtani A. S., Daghestani L. F., Ibrahim L. F. (2017). Environments and system types of virtual reality technology in STEM: a survey. *International Journal of Advanced Computer Science and Applications*.

[5] Zhang K., Suo J., Chen J., Xiaodi L., Lei G. Design and Implementation of Fire Safety Education System on Campus Based on Virtual Reality technology.

[6] Fernández R. P., Alonso V. (2015). Virtual Reality in a shipbuilding environment. *Advances in Engineering Software*.

[7] Gobe M. (2010). *Emotional Branding: The New Paradigm for Connecting Brands to people*.

[8] Hao P. (2016). On the Intrinsic Relationship between Regional Characteristics and Environmental Art Design. *International Conference on Education, Management, Computer and Society*.

[9] Schultz R. M., Williams C. J. (2002). The science of ART. *Science*.

[10] Wilson S. (2003). *Information Arts: Intersections of Art Science and technology*.

[11] Grau O. (2004). *Virtual Art: From Illusion to immersion*.

[12] Marxen E. (2009). Therapeutic thinking in contemporary art: or psychotherapy in the arts. *The Arts in Psychotherapy*.

[13] Jia Q., Keheng Z., Haiyang Y. (2017). Art design education in the new era featured with the integration of arts and motion sensing technology. *Eurasia Journal of Mathematics, Science and Technology Education*.

[14] Chang Y. S., Fang J. J. (2012). Revival of 1920s Belt-Driven Machine Tools with Virtual Reality. *Applied Mechanics and Materials*.

[15] Riva G., Baños R. M., Botella C., Wiederhold B. K., Gaggioli A. (2012). Positive technology: using interactive technologies to promote positive functioning. *Cyberpsychology, Behavior, and Social Networking*.

[16] McLellan H. (1996). Virtual realities. *Handbook of research for educational communications and technology*.

[17] Sutherland I. (1965). The Ultimate display. https://www.researchgate.net/publication/30875394_The_Ultimate_Display#citations.

[18] Dede C. (1996). The evolution of distance education: emerging technologies and distributed learning. *American Journal of Distance Education*.

[19] Defanti T. A., Brown M. D. (1991). Visualization in scientific computing. *Advances in Computers*.

[20] Papagiannakis G., Singh G., MagnenatThalmann N. (2008). A survey of mobile and wireless technologies for augmented reality systems. *Computer Animations and Virtual Worlds*.

[21] of Sciences N. A., of Engineering N. A. (2010). Efforts to avert the storm. *Rising Above the Gathering Storm, Revisited: Rapidly Approaching Category 5*.

[22] Su K. W., Chen S. C., Lin P. H., Hsieh C. I. (2020). Evaluating the user interface and experience of VR in the electronic commerce environment: a hybrid approach. *Virtual Reality*.

[23] Guo Z., Zhou D., Zhou Q. (2020). Applications of virtual reality in maintenance during the industrial product lifecycle: a systematic review. *Journal of Manufacturing Systems*.

[24] Earnshaw R. (2017). Art design and technology. *Collaboration and Implementation*.

[25] Gerber L. H., Narber C. G., Vishnoi N., Sidney L. (2014). The feasibility of using haptic devices to engage people with chronic traumatic brain injury in virtual 3D functional tasks. *Journal of NeuroEngineering and Rehabilitation*.

[26] Dalgarno B., Lee M. J. W. (2010). What are the learning affordances of 3D virtual environments?. *British Journal of Educational Technology*.

[27] Orr S., Shreeve A. (2017). Art and Design Pedagogy in Higher Education: Knowledge. *Values and Ambiguity in the Creative curriculum*.

[28] Zwirn S. G., Vande Zande R. (2017). Differences between art and design education—or differences in conceptions of creativity?. *Journal of Creative Behavior*.

[29] Danvers J. (2003). Towards a radical pedagogy: provisional notes on learning and teaching in art & design. *International Journal of Art and Design Education*.

[30] Jacob R. J. K., Karn K. S. (2003). *Eye tracking in human-computer interaction and usability research: ready to deliver the promises*.

[31] Serafin S., Erkut C., Kojs J., Nilsson N. C., Nordahl R. (2016). Virtual reality musical instruments: state of the art, design principles, and future directions. *Computer Music Journal*.

[32] Rizzo A. A., Buckwalter J. G. (1997). Virtual reality and cognitive assessment and rehabilitation: the state of the art. *Studies in Health Technology and Informatics*.

[33] Péruch P., Gaunet F., Thinus-Blanc C. (2018). Understanding and Learning Virtual spaces. *Cognitive Mapping*.

[34] Sanchez A., María Barreiro J., Maojo V. (2000). Design of virtual reality systems for education: a cognitive approach. *Education and Information Technologies*.

[35] Guo L., Wei D., Wang Y. (2021). Visual effect analysis of art design form based on visual psychology. *Psychiatria Danubina*.

